# Crystal structure of bis­(2-{[(pyridin-2-yl)methyl­idene]amino}­benzoato-κ^3^ *N*,*N*′,*O*)cobalt(II) *N*,*N*-di­methyl­formamide sesquisolvate

**DOI:** 10.1107/S1600536814019485

**Published:** 2014-09-06

**Authors:** Elena A. Buvaylo, Vladimir N. Kokozay, Olga Yu. Vassilyeva, Brian W. Skelton

**Affiliations:** a Department of Inorganic Chemistry, Taras Shevchenko National University of Kyiv, 64/13 Volodymyrska Street, Kyiv 01601, Ukraine; b Centre for Microscopy, Characterisation and Analysis, M313, University of Western Australia, Perth, WA 6009, Australia

**Keywords:** crystal structure, Co^II^ complex, Schiff base ligand, pyridine-2-carbaldehyde, anthranilic acid, hydrogen bonding

## Abstract

The title compound, [Co(C_13_H_9_N_2_O_2_)_2_]·1.5C_3_H_7_NO, is formed as a neutral Co^II^ complex with di­methyl­formamide (DMF) solvent mol­ecules. The Co^II^ atom has a distorted O_2_N_4_ octa­hedral coordination sphere defined by two tridentate anionic Schiff base ligands with the O atoms being *cis*. The coordination sphere around the Co^II^ atom is geometrically different from that reported for the co-crystal [Co(C_13_H_9_N_2_O_2_)_2_]·AA·H_2_O (AA is anthranilic acid). One of the DMF solvent mol­ecules was modelled as being disordered about a crystallographic inversion centre with half-occupancy. The crystal structure is made up from alternating layers of complex mol­ecules and DMF mol­ecules parallel to (010). C—H⋯O hydrogen-bonding inter­actions between the complex mol­ecules and the solvent mol­ecules consolidate the crystal packing.

## Abstract 2

The coordination geometry around the central Co^II^ ion is unexpectedly different from that for the co-crystal of the title mol­ecule with anthranilic acid.

## Chemical context   

Metal complexes containing Schiff bases are the most fundamental chelating systems in coordination chemistry. Their inter­esting chemical and physical properties and their wide-ranging applications in numerous scientific areas have been explored widely (Vigato *et al.*, 2012 ▶). During the last few years, we have investigated the chemistry of 3*d* metal complexes of Schiff base ligands with the aim of preparing mono- and heterometallic polynuclear compounds.
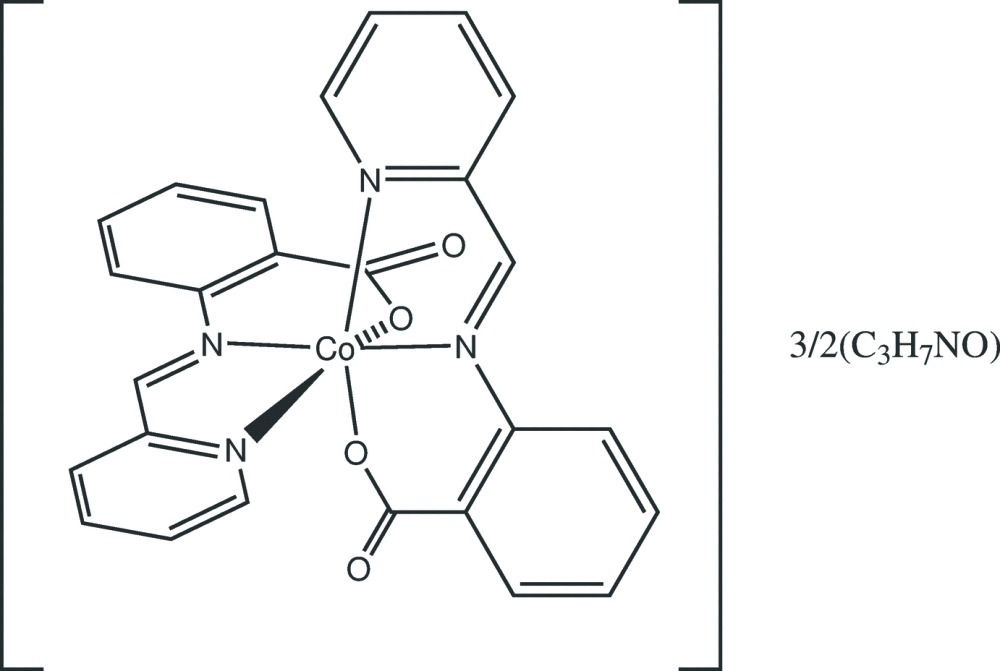


Recently, we have investigated the coordination behaviour of the tridentate carboxyl­ate Schiff base ligand 2-*N*-(2′-pyridyl­imine)­benzoic acid (H*L*), which results from the condensation between pyridine-2-carbaldehyde and anthran­ilic acid (AA) and reported the cation–anion complex Cr*L*_2_NO_3_·H_2_O (Buvaylo *et al.*, 2014*a* ▶) and co-crystals of *ML*_2_ (*M* = Co, Ni, Zn) and anthranilic acid (Buvaylo *et al.*, 2014*b* ▶). The respective compounds were prepared by *in situ* Schiff base synthesis. *ML*_2_ mol­ecules of the isotypic Co*L*_2_·AA·H_2_O and Ni*L*_2_·AA·H_2_O co-crystals retained the intra­molecular distances *M*—(N,O) as found in the structure of the ‘native’ Schiff base metal complex Ni*L*_2_·H_2_O (Mukhopadhyay & Pal, 2005 ▶). The crystal packing of the co-crystals was described as an insertion of the organic mol­ecules between the layers of *ML*_2_ complexes as they occur in the reported Ni*L*_2_·H_2_O structure.

The title compound, [Co(C_13_H_9_N_2_O_2_)_2_]·1.5C_3_H_7_NO, was prepared similarly to the co-crystals (Buvaylo *et al.*, 2014*b* ▶) but using additional [Cd(CH_3_COO)_2_]·2H_2_O in an attempt to prepare a heterometallic compound with H*L*. The obtained crystals, however, did not appear to contain anthranilic acid mol­ecules or cadmium.

## Structural commentary   

**Figure 1:**
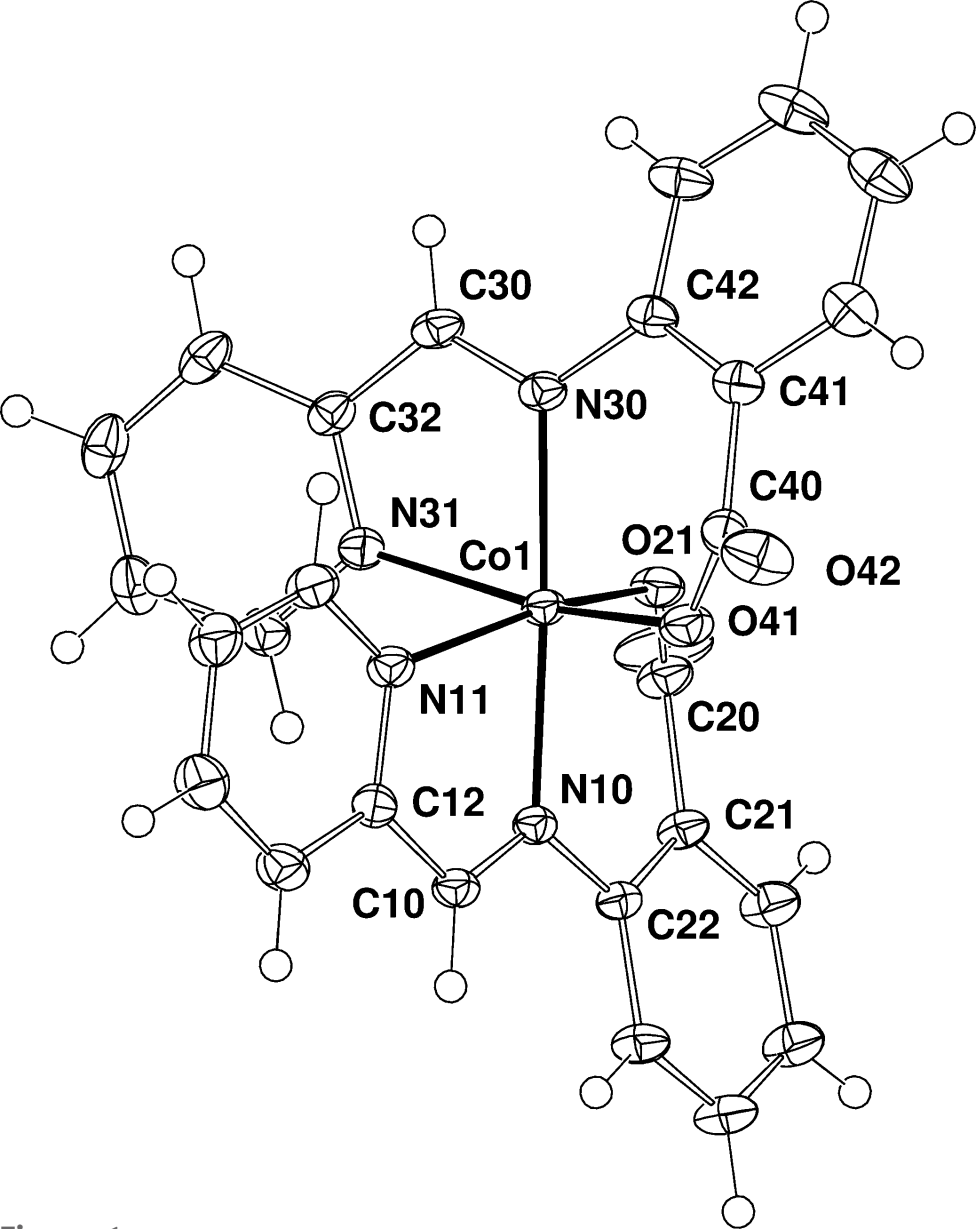
The mol­ecular structure of the title complex, showing the atom-numbering scheme. Non-H atoms are shown as displacement ellipsoids at the 50% probability level.

The asymmetric unit of the title compound consists of one neutral Co*L*_2_ mol­ecule and 1.5 di­methyl­formamide (DMF) solvent mol­ecules, of which one is fully ordered, the other being disordered about a crystallographic inversion centre. The Co*L*_2_ mol­ecule has no crystallographically imposed symmetry. The ligand mol­ecules are deprotonated at the carboxyl­ato oxygen atom and coordinate to the Co^II^ atom through the azomethine, pyridine-N and carboxyl­ato-O atoms in such a way that the metal atom is octa­hedrally surrounded by two anionic ligands with *cis* O atoms (Fig. 1 ▶, Table 1 ▶). The octa­hedral geometry is severely distorted: the Co—(N,O) distances fall in the range 2.0072 (12)–2.1498 (14) Å, the *trans* angles at the Co^II^ ion lie in the range 161.53 (6)–177.35 (5), the *cis* angles vary from 77.91 (5) to 103.70 (5)°. Surprisingly, the coordination geometry around the Co^II^ ion is markedly different from that of Co*L*_2_·AA·H_2_O (Buvaylo *et al.*, 2014*b* ▶) where the Co—(N,O) distances range from 1.990 (2) to 2.088 (18) Å, and the *trans* and *cis* angles at the Co^II^ ion vary from 167.96 (6) to 176.95 (7) and from 80.93 (7) to 98.81 (7)°, respectively. The reason for such a discrepancy could be the absence of classical hydrogen bonds in the title compound in contrast to the co-crystal Co*L*_2_·AA·H_2_O. A metal site with mixed (Co/Cd) occupancy for the title compound was ruled out by the refinement.

## Supra­molecular features   

**Figure 2:**
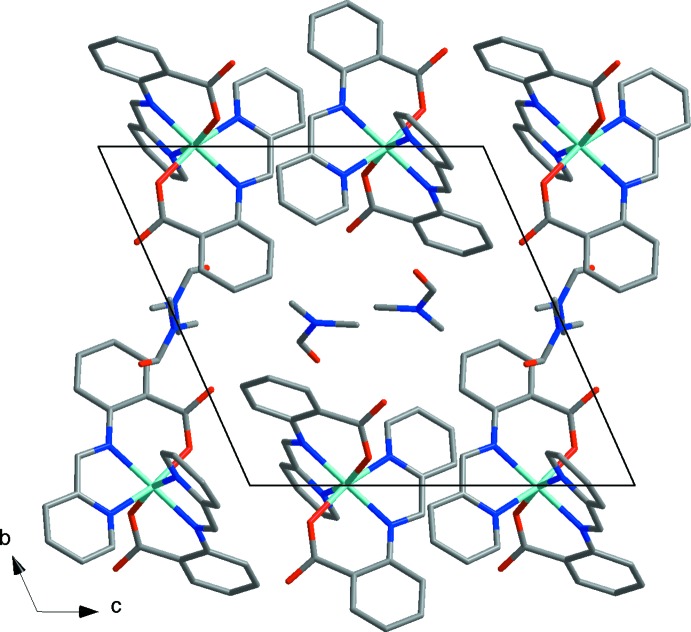
Packing diagram showing alternating layers of [Co*L*_2_] and DMF mol­ecules. CH hydrogens have been omitted for clarity.

The crystal lattice is built of alternating layers of complex Co*L*_2_ mol­ecules and DMF mol­ecules parallel to (010) (Fig. 2 ▶). Neighbouring Co*L*_2_ mol­ecules within a layer are related by an inversion centre with Co⋯Co separations of 6.8713 (6) and 6.9985 (6) Å. Weak C—H⋯O hydrogen-bonding inter­actions between the complex mol­ecules and the solvent mol­ecules lead to a consolidation of the crystal packing.

## Synthesis and crystallization   

The Schiff base ligand H*L* was prepared by refluxing pyridine-2-carbaldehyde (0.38 ml, 4 mmol) with anthranilic acid (0.55 g, 4 mmol) in 20 ml methanol for half an hour. The resultant yellow solution was left in open air overnight and used without further purification.

To a stirred DMF solution (5 ml) of Cd(CH_3_COO)_2_·2H_2_O (0.53 g, 2 mmol) in a 50 ml conic flask, H*L* (0.21 g, 4 mmol) in methanol from the previous preparation was added. The solution was magnetically stirred at 323 K for 20 minutes and a yellow precipitate of a Cd complex formed. Co(CH_3_COO)_2_·4H_2_O (0.25 g, 1 mmol) in DMF (10 ml) was added to the reaction mixture after a week. The mixture was stirred magnetically at 323 K for an hour, however, the yellow precipitate did not dissolve and was filtered off. The resulting red–brown solution was left to evaporate at room temperature. Red–brown block-like crystals of the title compound formed the next day. They were collected by filter-suction, washed with dry iso­propanol and finally dried *in vacuo* (yield: 23% based on cobalt salt). Analysis for C_26_H_18_CoN_4_O_4_·1.5C_3_H_7_NO calculated (%) C: 59.18 H: 4.64 N: 12.45 Co: 9.52. Found (%) C: 59.33 H: 4.49 N: 12.41 Co: 9.76. Spectroscopic data (IR, KBr) are available as an additional Figure in the supporting information.

## Refinement   

Crystal data, data collection and structure refinement details are summarized in Table 3 ▶. The refinement of the metal occupancy as part Co and part Cd gave 100% Co. One solvent DMF mol­ecule was modelled as being disordered about a crystallographic inversion centre with resulting half-occupancy and with geometries restrained to ideal values. All hydrogen atoms were placed at calculated positions and refined by use of the riding-model approximation, with *U*_iso_(H) = 1.2*U*_eq_ of the parent C atom.

## Supplementary Material

Crystal structure: contains datablock(s) I, global. DOI: 10.1107/S1600536814019485/wm5044sup1.cif

Structure factors: contains datablock(s) I. DOI: 10.1107/S1600536814019485/wm5044Isup2.hkl

Supporting information file. DOI: 10.1107/S1600536814019485/wm5044Isup3.jpg

CCDC reference: 1021534

Additional supporting information:  crystallographic information; 3D view; checkCIF report

## Figures and Tables

**Table 1:** Selected bond lengths (Å)

Co1—O41	2.0072 (12)	Co1—N10	2.1189 (13)
Co1—O21	2.0181 (13)	Co1—N31	2.1358 (14)
Co1—N30	2.1057 (13)	Co1—N11	2.1498 (14)

**Table 2:** Hydrogen-bond geometry (Å, °)

*D*—H⋯*A*	*D*—H	H⋯*A*	*D*⋯*A*	*D*—H⋯*A*
C10—H10⋯O42^i^	0.95	2.46	3.393 (2)	169
C102—H10*E*⋯O42^ii^	0.98	2.46	3.369 (3)	154
C16—H16⋯O101	0.95	2.41	3.326 (3)	163
C201—H20*C*⋯O22^iii^	0.98	1.97	2.819 (10)	143
C23—H23⋯O42^i^	0.95	2.60	3.454 (2)	150
C30—H30⋯O22^iv^	0.95	2.42	3.344 (3)	163
C36—H36⋯O201^v^	0.95	2.54	3.235 (13)	130

Symmetry codes: (i) 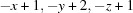; (ii) 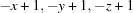; (iii) 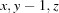; (iv) 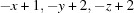; (v) 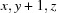.

**Table 3:** Experimental details

Crystal data	
Chemical formula	[Co(C_13_H_9_N_2_O_2_)_2_]·1.5C_3_H_7_NO
*M*_r_	619.02
Crystal system, space group	Triclinic, *P* 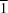
Temperature (K)	100
*a*, *b*, *c* (Å)	8.4361 (6), 13.2603 (10), 13.8664 (10)
α, β, γ (°)	110.061 (7), 103.559 (6), 101.430 (6)
*V* (Å^3^)	1348.9 (2)
*Z*	2
Radiation type	Mo *K*α
μ (mm^−1^)	0.69
Crystal size (mm)	0.40 × 0.30 × 0.18

Data collection	
Diffractometer	Oxford Diffraction Xcalibur
Absorption correction	Analytical [*CrysAlis PRO* (Agilent, 2011 ▶) using an expression derived by Clark & Reid (1995 ▶)]
*T*_min_, *T*_max_	0.821, 0.898
No. of measured, independent and observed [*I* > 2σ(*I*)] reflections	33209, 10748, 8599
*R*_int_	0.036
(sin θ/λ)_max_ (Å^−1^)	0.787

Refinement	
*R*[*F* ^2^ > 2σ(*F* ^2^)], *wR*(*F* ^2^), *S*	0.048, 0.127, 1.05
No. of reflections	10748
No. of parameters	410
No. of restraints	35
H-atom treatment	H-atom parameters constrained
Δρ_max_, Δρ_min_ (e Å^−3^)	0.74, −0.54

Computer programs: *CrysAlis PRO* (Agilent, 2011 ▶), *SIR92* (Altomare *et al.*, 1994 ▶), *SHELXL97* (Sheldrick, 2008 ▶), *ORTEPII* (Johnson, 1976 ▶), *DIAMOND* (Brandenburg, 1999 ▶) and *publCIF* (Westrip, 2010 ▶).

## supplementary crystallographic information

### Crystal data

**Table:**

[Co(C_13_H_9_N_2_O_2_)_2_]·1.5C_3_H_7_NO	*Z* = 2
*M**_r_* = 619.02	*F*(000) = 642
Triclinic, *P*1	*D*_x_ = 1.524 Mg m^−^^3^
Hall symbol: -P 1	Mo *K*α radiation, λ = 0.71073 Å
*a* = 8.4361 (6) Å	Cell parameters from 10672 reflections
*b* = 13.2603 (10) Å	θ = 2.8–34.5°
*c* = 13.8664 (10) Å	µ = 0.69 mm^−^^1^
α = 110.061 (7)°	*T* = 100 K
β = 103.559 (6)°	Block, red-brown
γ = 101.430 (6)°	0.40 × 0.30 × 0.18 mm
*V* = 1348.9 (2) Å^3^	

### Data collection

**Table:**

Oxford Diffraction Xcalibur diffractometer	10748 independent reflections
Graphite monochromator	8599 reflections with *I* > 2σ(*I*)
Detector resolution: 16.0009 pixels mm^-1^	*R*_int_ = 0.036
ω scans	θ_max_ = 34°, θ_min_ = 2.8°
Absorption correction: analytical [*CrysAlis PRO* (Agilent, 2011) using an expression derived by Clark & Reid (1995)]	*h* = −12→13
*T*_min_ = 0.821, *T*_max_ = 0.898	*k* = −20→19
33209 measured reflections	*l* = −21→21

### Refinement

**Table:**

Refinement on *F*^2^	Primary atom site location: structure-invariant direct methods
Least-squares matrix: full	Secondary atom site location: difference Fourier map
*R*[*F*^2^ > 2σ(*F*^2^)] = 0.048	Hydrogen site location: inferred from neighbouring sites
*wR*(*F*^2^) = 0.127	H-atom parameters constrained
*S* = 1.05	*w* = 1/[σ^2^(*F*_o_^2^) + (0.0594*P*)^2^ + 0.4745*P*] where *P* = (*F*_o_^2^ + 2*F*_c_^2^)/3
10748 reflections	(Δ/σ)_max_ = 0.006
410 parameters	Δρ_max_ = 0.74 e Å^−^^3^
35 restraints	Δρ_min_ = −0.54 e Å^−^^3^

### Special details

**Table:**

Geometry. All s.u.'s (except the s.u. in the dihedral angle between two l.s. planes) are estimated using the full covariance matrix. The cell s.u.'s are taken into account individually in the estimation of s.u.'s in distances, angles and torsion angles; correlations between s.u.'s in cell parameters are only used when they are defined by crystal symmetry. An approximate (isotropic) treatment of cell s.u.'s is used for estimating s.u.'s involving l.s. planes.
Refinement. Refinement of *F*^2^ against ALL reflections. The weighted *R*-factor *wR* and goodness of fit *S* are based on *F*^2^, conventional *R*-factors *R* are based on *F*, with *F* set to zero for negative *F*^2^. The threshold expression of *F*^2^ > 2σ(*F*^2^) is used only for calculating *R*-factors(gt) *etc*. and is not relevant to the choice of reflections for refinement. *R*-factors based on *F*^2^ are statistically about twice as large as those based on *F*, and *R*- factors based on ALL data will be even larger.One solvent dmf molecule was modelled as being disordered about a crystallographic inversion centre. The geometries were restrained to ideal values.

### Fractional atomic coordinates and isotropic or equivalent isotropic displacement parameters (Å^2^)

**Table:**

	*x*	*y*	*z*	*U*_iso_*/*U*_eq_	Occ. (<1)
Co1	0.50566 (3)	0.996180 (17)	0.747260 (16)	0.01696 (6)	
N11	0.28320 (17)	0.91486 (12)	0.60369 (11)	0.0197 (2)	
C12	0.2706 (2)	0.97508 (14)	0.54251 (12)	0.0194 (3)	
C13	0.1383 (2)	0.93697 (15)	0.44446 (14)	0.0255 (3)	
H13	0.1345	0.9792	0.4012	0.031*	
C14	0.0121 (2)	0.83562 (17)	0.41152 (15)	0.0307 (4)	
H14	−0.0801	0.8077	0.3453	0.037*	
C15	0.0221 (2)	0.77579 (16)	0.47596 (15)	0.0289 (4)	
H15	−0.0645	0.7073	0.4556	0.035*	
C16	0.1607 (2)	0.81754 (15)	0.57109 (14)	0.0245 (3)	
H16	0.1687	0.7755	0.6144	0.029*	
C10	0.4017 (2)	1.08481 (13)	0.58651 (12)	0.0192 (3)	
H10	0.4017	1.131	0.5472	0.023*	
N10	0.51714 (16)	1.11742 (11)	0.67961 (10)	0.0170 (2)	
C21	0.7536 (2)	1.26504 (14)	0.83458 (12)	0.0205 (3)	
C22	0.65025 (19)	1.22222 (13)	0.72478 (12)	0.0174 (3)	
C23	0.6816 (2)	1.28292 (14)	0.66229 (13)	0.0240 (3)	
H23	0.6106	1.2552	0.5888	0.029*	
C24	0.8144 (2)	1.38261 (15)	0.70634 (14)	0.0277 (3)	
H24	0.833	1.4234	0.6634	0.033*	
C25	0.9208 (2)	1.42328 (16)	0.81350 (15)	0.0285 (4)	
H25	1.0149	1.4901	0.8431	0.034*	
C26	0.8883 (2)	1.36573 (15)	0.87639 (14)	0.0269 (3)	
H26	0.9591	1.3952	0.9501	0.032*	
C20	0.7319 (2)	1.21453 (15)	0.91605 (13)	0.0240 (3)	
O21	0.69029 (16)	1.10880 (10)	0.88645 (9)	0.0232 (2)	
O22	0.7636 (3)	1.28251 (13)	1.01092 (11)	0.0475 (4)	
N31	0.31332 (17)	1.02612 (12)	0.82016 (11)	0.0203 (2)	
C32	0.2642 (2)	0.94913 (14)	0.85884 (12)	0.0197 (3)	
C33	0.1204 (2)	0.93994 (16)	0.89184 (13)	0.0257 (3)	
H33	0.0913	0.8875	0.9222	0.031*	
C34	0.0202 (2)	1.00887 (17)	0.87943 (13)	0.0284 (4)	
H34	−0.0811	1.0023	0.8986	0.034*	
C35	0.0694 (2)	1.08726 (17)	0.83884 (13)	0.0270 (3)	
H35	0.0025	1.1352	0.8296	0.032*	
C36	0.2188 (2)	1.09467 (16)	0.81177 (13)	0.0245 (3)	
H36	0.255	1.1504	0.7864	0.029*	
C30	0.3667 (2)	0.87175 (14)	0.85948 (12)	0.0205 (3)	
H30	0.3484	0.821	0.8931	0.025*	
N30	0.48205 (17)	0.87471 (11)	0.81309 (10)	0.0181 (2)	
C41	0.6719 (2)	0.78101 (13)	0.73476 (12)	0.0207 (3)	
C42	0.5915 (2)	0.80600 (13)	0.81452 (12)	0.0199 (3)	
C43	0.6270 (2)	0.76728 (15)	0.89722 (14)	0.0271 (3)	
H43	0.5707	0.7825	0.9501	0.033*	
C44	0.7429 (3)	0.70727 (16)	0.90256 (15)	0.0329 (4)	
H44	0.7668	0.6823	0.9594	0.039*	
C45	0.8246 (3)	0.68328 (15)	0.82488 (15)	0.0313 (4)	
H45	0.9061	0.6434	0.8293	0.038*	
C46	0.7858 (2)	0.71809 (14)	0.74084 (14)	0.0261 (3)	
H46	0.838	0.6987	0.6861	0.031*	
C40	0.6515 (2)	0.81957 (14)	0.64211 (13)	0.0214 (3)	
O41	0.63573 (16)	0.91724 (11)	0.65820 (10)	0.0244 (2)	
O42	0.6581 (2)	0.75447 (12)	0.55549 (10)	0.0344 (3)	
C101	0.2797 (3)	0.5260 (2)	0.53693 (17)	0.0462 (6)	
H10A	0.1781	0.5472	0.5112	0.069*	
H10B	0.2719	0.4523	0.4839	0.069*	
H10C	0.3825	0.5826	0.5457	0.069*	
C102	0.4048 (3)	0.4635 (2)	0.6791 (2)	0.0492 (6)	
H10D	0.5229	0.5046	0.6912	0.074*	
H10E	0.3748	0.3864	0.6245	0.074*	
H10F	0.3947	0.4615	0.7475	0.074*	
N101	0.2892 (2)	0.52022 (14)	0.64083 (13)	0.0314 (3)	
C103	0.2097 (2)	0.57619 (16)	0.70349 (15)	0.0292 (4)	
H103	0.2267	0.5737	0.7728	0.035*	
O101	0.1172 (2)	0.63017 (13)	0.68000 (13)	0.0384 (3)	
C201	0.6583 (8)	0.4774 (8)	1.0471 (4)	0.074 (2)	0.5
H20A	0.6872	0.5372	1.1196	0.11*	0.5
H20B	0.7402	0.4981	1.0122	0.11*	0.5
H20C	0.6635	0.4067	1.0537	0.11*	0.5
C202	0.4086 (11)	0.5472 (7)	1.0070 (9)	0.094 (3)	0.5
H20D	0.4843	0.62	1.0193	0.141*	0.5
H20E	0.3758	0.5541	1.0718	0.141*	0.5
H20F	0.3056	0.5245	0.9445	0.141*	0.5
N201	0.4968 (10)	0.4635 (6)	0.9856 (6)	0.0754 (17)	0.5
C203	0.4235 (7)	0.3681 (6)	0.9046 (5)	0.0535 (12)	0.5
H203	0.4778	0.312	0.8829	0.064*	0.5
O201	0.2772 (15)	0.3581 (9)	0.8592 (9)	0.211 (4)	0.5

### Atomic displacement parameters (Å^2^)

**Table:**

	*U*^11^	*U*^22^	*U*^33^	*U*^12^	*U*^13^	*U*^23^
Co1	0.01868 (10)	0.01863 (11)	0.01563 (10)	0.00464 (7)	0.00591 (7)	0.00978 (8)
N11	0.0208 (6)	0.0218 (6)	0.0165 (5)	0.0045 (5)	0.0054 (5)	0.0092 (5)
C12	0.0192 (6)	0.0232 (7)	0.0163 (6)	0.0063 (5)	0.0056 (5)	0.0091 (5)
C13	0.0258 (8)	0.0286 (8)	0.0208 (7)	0.0062 (6)	0.0038 (6)	0.0123 (6)
C14	0.0247 (8)	0.0347 (10)	0.0249 (8)	0.0029 (7)	−0.0012 (6)	0.0127 (7)
C15	0.0231 (8)	0.0294 (9)	0.0267 (8)	−0.0005 (6)	0.0024 (6)	0.0112 (7)
C16	0.0243 (7)	0.0252 (8)	0.0224 (7)	0.0019 (6)	0.0055 (6)	0.0124 (6)
C10	0.0223 (7)	0.0213 (7)	0.0171 (6)	0.0071 (5)	0.0075 (5)	0.0107 (5)
N10	0.0191 (6)	0.0183 (6)	0.0157 (5)	0.0061 (5)	0.0067 (4)	0.0084 (5)
C21	0.0247 (7)	0.0207 (7)	0.0176 (6)	0.0041 (6)	0.0087 (5)	0.0102 (5)
C22	0.0207 (6)	0.0172 (6)	0.0170 (6)	0.0059 (5)	0.0082 (5)	0.0087 (5)
C23	0.0317 (8)	0.0237 (8)	0.0182 (7)	0.0058 (6)	0.0082 (6)	0.0120 (6)
C24	0.0372 (9)	0.0241 (8)	0.0243 (8)	0.0040 (7)	0.0110 (7)	0.0149 (7)
C25	0.0335 (9)	0.0239 (8)	0.0271 (8)	0.0017 (7)	0.0096 (7)	0.0133 (7)
C26	0.0305 (8)	0.0235 (8)	0.0213 (7)	−0.0004 (6)	0.0046 (6)	0.0102 (6)
C20	0.0273 (8)	0.0249 (8)	0.0182 (7)	0.0017 (6)	0.0070 (6)	0.0108 (6)
O21	0.0260 (6)	0.0228 (6)	0.0201 (5)	0.0040 (4)	0.0039 (4)	0.0123 (4)
O22	0.0860 (13)	0.0276 (7)	0.0186 (6)	−0.0018 (8)	0.0160 (7)	0.0088 (5)
N31	0.0203 (6)	0.0258 (7)	0.0176 (6)	0.0068 (5)	0.0067 (5)	0.0118 (5)
C32	0.0200 (7)	0.0224 (7)	0.0140 (6)	0.0021 (5)	0.0055 (5)	0.0068 (5)
C33	0.0246 (8)	0.0298 (8)	0.0192 (7)	0.0005 (6)	0.0102 (6)	0.0082 (6)
C34	0.0200 (7)	0.0399 (10)	0.0181 (7)	0.0039 (7)	0.0076 (6)	0.0056 (7)
C35	0.0238 (8)	0.0385 (10)	0.0179 (7)	0.0132 (7)	0.0063 (6)	0.0089 (7)
C36	0.0252 (8)	0.0333 (9)	0.0208 (7)	0.0130 (7)	0.0092 (6)	0.0143 (7)
C30	0.0245 (7)	0.0208 (7)	0.0145 (6)	0.0023 (6)	0.0056 (5)	0.0086 (5)
N30	0.0202 (6)	0.0179 (6)	0.0139 (5)	0.0025 (5)	0.0033 (4)	0.0071 (4)
C41	0.0233 (7)	0.0172 (7)	0.0171 (6)	0.0034 (5)	0.0027 (5)	0.0060 (5)
C42	0.0233 (7)	0.0165 (6)	0.0158 (6)	0.0035 (5)	0.0019 (5)	0.0063 (5)
C43	0.0380 (9)	0.0236 (8)	0.0184 (7)	0.0082 (7)	0.0044 (6)	0.0108 (6)
C44	0.0454 (11)	0.0269 (9)	0.0228 (8)	0.0134 (8)	0.0002 (7)	0.0121 (7)
C45	0.0380 (10)	0.0210 (8)	0.0285 (8)	0.0120 (7)	0.0002 (7)	0.0082 (7)
C46	0.0278 (8)	0.0207 (7)	0.0236 (7)	0.0071 (6)	0.0033 (6)	0.0056 (6)
C40	0.0207 (7)	0.0247 (8)	0.0191 (7)	0.0066 (6)	0.0062 (5)	0.0098 (6)
O41	0.0304 (6)	0.0269 (6)	0.0244 (6)	0.0114 (5)	0.0145 (5)	0.0153 (5)
O42	0.0531 (9)	0.0341 (7)	0.0206 (6)	0.0208 (7)	0.0145 (6)	0.0107 (5)
C101	0.0517 (13)	0.0484 (13)	0.0279 (9)	0.0014 (11)	0.0207 (10)	0.0063 (9)
C102	0.0334 (11)	0.0434 (13)	0.0490 (13)	0.0134 (10)	0.0020 (10)	0.0004 (11)
N101	0.0294 (8)	0.0303 (8)	0.0251 (7)	0.0033 (6)	0.0089 (6)	0.0035 (6)
C103	0.0306 (9)	0.0291 (9)	0.0251 (8)	0.0031 (7)	0.0119 (7)	0.0094 (7)
O101	0.0432 (8)	0.0358 (8)	0.0400 (8)	0.0142 (7)	0.0170 (7)	0.0165 (7)
C201	0.036 (3)	0.148 (6)	0.030 (2)	0.033 (3)	0.004 (2)	0.031 (3)
C202	0.055 (4)	0.078 (5)	0.136 (6)	0.025 (4)	0.035 (5)	0.024 (5)
N201	0.049 (2)	0.115 (4)	0.071 (3)	0.016 (4)	0.020 (3)	0.053 (4)
C203	0.043 (3)	0.079 (4)	0.056 (3)	0.033 (3)	0.019 (2)	0.039 (3)
O201	0.227 (7)	0.144 (6)	0.183 (7)	0.017 (6)	0.015 (6)	0.032 (6)

### Geometric parameters (Å, º)

**Table:**

Co1—O41	2.0072 (12)	C35—C36	1.392 (2)
Co1—O21	2.0181 (13)	C35—H35	0.9500
Co1—N30	2.1057 (13)	C36—H36	0.9500
Co1—N10	2.1189 (13)	C30—N30	1.288 (2)
Co1—N31	2.1358 (14)	C30—H30	0.9500
Co1—N11	2.1498 (14)	N30—C42	1.421 (2)
N11—C16	1.338 (2)	C41—C46	1.398 (2)
N11—C16	1.338 (2)	C41—C42	1.409 (2)
N11—C12	1.351 (2)	C41—C40	1.524 (2)
C12—C13	1.394 (2)	C42—C43	1.404 (2)
C12—C10	1.466 (2)	C43—C44	1.382 (3)
C13—C14	1.390 (3)	C43—H43	0.9500
C13—H13	0.9500	C44—C45	1.392 (3)
C14—C15	1.383 (3)	C44—H44	0.9500
C14—H14	0.9500	C45—C46	1.389 (3)
C15—C16	1.390 (2)	C45—H45	0.9500
C15—H15	0.9500	C46—H46	0.9500
C16—H16	0.9500	C40—O42	1.237 (2)
C10—N10	1.289 (2)	C40—O41	1.276 (2)
C10—H10	0.9500	C101—N101	1.453 (3)
N10—C22	1.428 (2)	C101—H10A	0.9800
C21—C26	1.403 (2)	C101—H10B	0.9800
C21—C22	1.410 (2)	C101—H10C	0.9800
C21—C20	1.525 (2)	C102—N101	1.456 (3)
C22—C23	1.404 (2)	C102—H10D	0.9800
C23—C24	1.384 (2)	C102—H10E	0.9800
C23—H23	0.9500	C102—H10F	0.9800
C24—C25	1.392 (3)	N101—C103	1.336 (2)
C24—H24	0.9500	C103—O101	1.221 (2)
C25—C26	1.380 (2)	C103—H103	0.9500
C25—H25	0.9500	C201—N201	1.366 (9)
C26—H26	0.9500	C201—H20A	0.9800
C20—O22	1.241 (2)	C201—H20B	0.9800
C20—O21	1.265 (2)	C201—H20C	0.9800
N31—C36	1.339 (2)	C202—N201	1.442 (10)
N31—C32	1.347 (2)	C202—H20D	0.9800
C32—C33	1.391 (2)	C202—H20E	0.9800
C32—C30	1.468 (2)	C202—H20F	0.9800
C33—C34	1.387 (3)	N201—C203	1.278 (9)
C33—H33	0.9500	C203—O201	1.204 (11)
C34—C35	1.382 (3)	C203—H203	0.9500
C34—H34	0.9500		
			
O41—Co1—O21	103.70 (5)	C33—C32—C30	121.74 (15)
O41—Co1—N30	89.97 (5)	C34—C33—C32	118.64 (16)
O21—Co1—N30	90.57 (5)	C34—C33—H33	120.7
O41—Co1—N10	91.81 (5)	C32—C33—H33	120.7
O21—Co1—N10	90.92 (5)	C35—C34—C33	119.31 (15)
N30—Co1—N10	177.35 (5)	C35—C34—H34	120.3
O41—Co1—N31	161.53 (6)	C33—C34—H34	120.3
O21—Co1—N31	90.55 (5)	C34—C35—C36	118.72 (17)
N30—Co1—N31	78.03 (5)	C34—C35—H35	120.6
N10—Co1—N31	99.76 (5)	C36—C35—H35	120.6
O41—Co1—N11	87.74 (5)	N31—C36—C35	122.40 (16)
O21—Co1—N11	164.36 (5)	N31—C36—H36	118.8
N30—Co1—N11	100.20 (5)	C35—C36—H36	118.8
N10—Co1—N11	77.91 (5)	N30—C30—C32	118.03 (14)
N31—Co1—N11	80.75 (5)	N30—C30—H30	121.0
C16—N11—C12	118.76 (14)	C32—C30—H30	121.0
C16—N11—Co1	128.75 (11)	C30—N30—C42	121.33 (14)
C12—N11—Co1	112.47 (10)	C30—N30—Co1	114.82 (11)
N11—C12—C13	122.22 (15)	C42—N30—Co1	123.70 (10)
N11—C12—C10	116.19 (13)	C46—C41—C42	118.51 (15)
C13—C12—C10	121.57 (15)	C46—C41—C40	115.46 (15)
C14—C13—C12	118.33 (16)	C42—C41—C40	126.01 (15)
C14—C13—H13	120.8	C43—C42—C41	119.54 (16)
C12—C13—H13	120.8	C43—C42—N30	120.99 (15)
C15—C14—C13	119.39 (16)	C41—C42—N30	119.40 (14)
C15—C14—H14	120.3	C44—C43—C42	120.71 (17)
C13—C14—H14	120.3	C44—C43—H43	119.6
C14—C15—C16	118.94 (17)	C42—C43—H43	119.6
C14—C15—H15	120.5	C43—C44—C45	120.18 (17)
C16—C15—H15	120.5	C43—C44—H44	119.9
N11—C16—C15	122.28 (16)	C45—C44—H44	119.9
N11—C16—H16	118.9	C46—C45—C44	119.41 (17)
C15—C16—H16	118.9	C46—C45—H45	120.3
N10—C10—C12	118.75 (14)	C44—C45—H45	120.3
N10—C10—H10	120.6	C45—C46—C41	121.60 (17)
C12—C10—H10	120.6	C45—C46—H46	119.2
C10—N10—C22	120.97 (13)	C41—C46—H46	119.2
C10—N10—Co1	114.38 (11)	O42—C40—O41	123.49 (15)
C22—N10—Co1	124.31 (10)	O42—C40—C41	116.99 (15)
C26—C21—C22	118.32 (14)	O41—C40—C41	119.46 (14)
C26—C21—C20	115.13 (14)	C40—O41—Co1	127.35 (11)
C22—C21—C20	126.52 (14)	N101—C101—H10A	109.5
C23—C22—C21	119.32 (14)	N101—C101—H10B	109.5
C23—C22—N10	121.59 (14)	H10A—C101—H10B	109.5
C21—C22—N10	119.08 (13)	N101—C101—H10C	109.5
C24—C23—C22	120.89 (15)	H10A—C101—H10C	109.5
C24—C23—H23	119.6	H10B—C101—H10C	109.5
C22—C23—H23	119.6	N101—C102—H10D	109.5
C23—C24—C25	120.10 (15)	N101—C102—H10E	109.5
C23—C24—H24	120.0	H10D—C102—H10E	109.5
C25—C24—H24	120.0	N101—C102—H10F	109.5
C26—C25—C24	119.40 (17)	H10D—C102—H10F	109.5
C26—C25—H25	120.3	H10E—C102—H10F	109.5
C24—C25—H25	120.3	C103—N101—C101	120.47 (19)
C25—C26—C21	121.91 (16)	C103—N101—C102	121.65 (18)
C25—C26—H26	119.0	C101—N101—C102	117.44 (19)
C21—C26—H26	119.0	O101—C103—N101	126.04 (18)
O22—C20—O21	123.53 (15)	O101—C103—H103	117.0
O22—C20—C21	116.35 (15)	N101—C103—H103	117.0
O21—C20—C21	120.06 (14)	C203—N201—C201	115.3 (7)
C20—O21—Co1	124.57 (11)	C203—N201—C202	120.1 (8)
C36—N31—C32	118.68 (14)	C201—N201—C202	124.6 (7)
C36—N31—Co1	127.42 (11)	O201—C203—N201	111.9 (8)
C32—N31—Co1	112.43 (11)	O201—C203—H203	124.1
N31—C32—C33	122.15 (16)	N201—C203—H203	124.1
N31—C32—C30	116.05 (13)		

### Hydrogen-bond geometry (Å, º)

**Table:**

*D*—H···*A*	*D*—H	H···*A*	*D*···*A*	*D*—H···*A*
C10—H10···O42^i^	0.95	2.46	3.393 (2)	169
C102—H10*E*···O42^ii^	0.98	2.46	3.369 (3)	154
C16—H16···O101	0.95	2.41	3.326 (3)	163
C201—H20*C*···O22^iii^	0.98	1.97	2.819 (10)	143
C23—H23···O42^i^	0.95	2.60	3.454 (2)	150
C30—H30···O22^iv^	0.95	2.42	3.344 (3)	163
C36—H36···O201^v^	0.95	2.54	3.235 (13)	130

Symmetry codes: (i) −*x*+1, −*y*+2, −*z*+1; (ii) −*x*+1, −*y*+1, −*z*+1; (iii) *x*, *y*−1, *z*; (iv) −*x*+1, −*y*+2, −*z*+2; (v) *x*, *y*+1, *z*.
