# Supplementary figures and images for: Crystal structure of bis­(2-{[(pyridin-2-yl)methyl­idene]amino}­benzoato-κ3 N,N′,O)cobalt(II) N,N-di­methyl­formamide sesquisolvate
Source: Acta Crystallogr Sect E Struct Rep Online. 2014 Sep 6;70(Pt 10):164–6. doi: 10.1107/S1600536814019485 (PMC4257231; doi:10.1107/S1600536814019485)

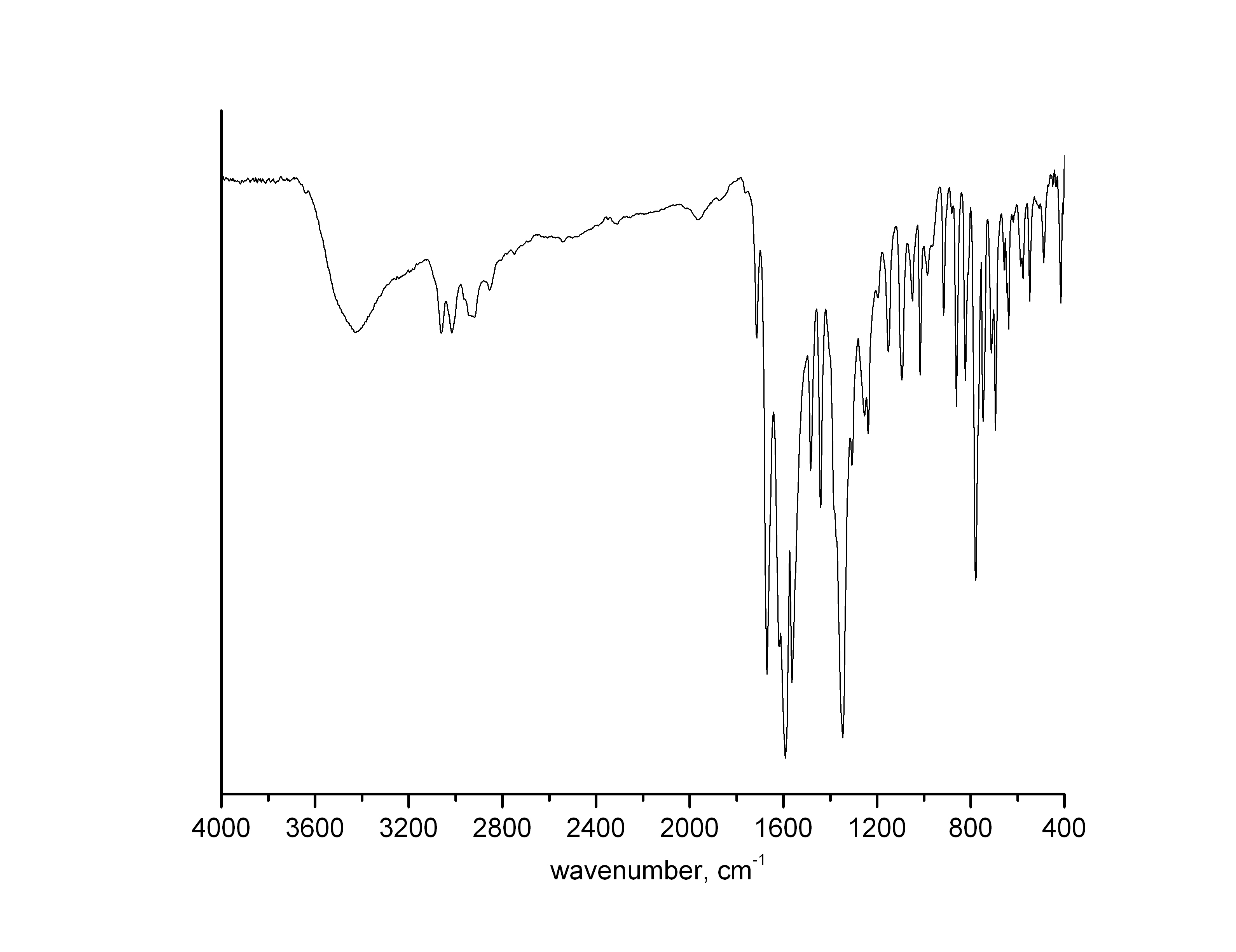

Supplement: Supplementary file 3 [file e-70-00164-Isup3.jpg]
